# The condensin complexes play distinct roles to ensure normal chromosome morphogenesis during meiotic division in Arabidopsis

**DOI:** 10.1111/tpj.12628

**Published:** 2014-07-26

**Authors:** Sarah J Smith, Kim Osman, F Christopher H Franklin

**Affiliations:** 1 School of Biosciences, University of BirminghamEdgbaston, Birmingham, B15 2TT, UK; 2 School of Biological and Biomedical Sciences, Durham UniversitySouth Road, Durham, DH1 3LE, UK

**Keywords:** condensin complex, meiosis, chromosome segregation, recombination, *Arabidopsis thaliana*

## Abstract

Meiosis is a specialized cell division essential for sexual reproduction. During meiosis the chromosomes are highly organized, and correct chromosome architecture is required for faithful segregation of chromosomes at anaphase I and II. Condensin is involved in chromosome organization during meiotic and mitotic cell divisions. Three condensin subunits, *At*SMC4 and the condensin I and II specific subunits *At*CAP-D2 and *At*CAP-D3, respectively, have been studied for their role in meiosis. This has revealed that both the condensin I and condensin II complexes are required to maintain normal structural integrity of the meiotic chromosomes during the two nuclear divisions. Their roles appear functionally distinct in that condensin I is required to maintain normal compaction of the centromeric repeats and *45S* rDNA, whereas loss of condensin II was associated with extensive interchromosome connections at metaphase I. Depletion of condensin is also associated with a slight reduction in crossover formation, suggesting a role during meiotic prophase I.

## Introduction

During mitotic and meiotic cell divisions chromosomes undergo massive condensation from interphase to metaphase. This compaction of the chromosomes is essential for the accurate segregation of chromosomes at anaphase. The underlying mechanism is largely unknown, and in particular the structural arrangement of the metaphase chromosomes remains elusive. One protein complex that has been identified as a key player in chromosome organization is condensin. Condensin is a pentameric complex that comprises two members of the structural maintenance of chromosomes (SMC) family, SMC2 and SMC4, as well as three non-SMC regulatory subunits. In higher eukaryotes two forms of condensin complex exist. Both contain the SMC2 and SMC4 backbone, but have different regulatory proteins. Condensin I comprises CAP-H/Barren, CAP-D2 and CAP-G, whereas condensin II comprises CAP-H2, CAP-D3 and CAP-G2 (Schmiesing *et al*., 1998; Ono *et al*., 2003; Yeong *et al*., 2003; Hirano, 2005). The two complexes appear to play distinct roles in chromosome organization, as reducing levels of each complex individually has different effects on the shape of the chromosomes (Ono *et al*., 2003). Some studies suggest that condensin II may have an earlier role in mitotic chromosome condensation than condensin I (Ono *et al*., 2003, 2004; Hirota *et al*., 2004; Gerlich *et al*., 2006).

Initial studies of condensin in mitotic cells indicated that the complex was required for chromosome condensation (Saka *et al*., 1994; Strunnikov *et al*., 1995; Hirano *et al*., 1997; Sutani *et al*., 1999; Freeman *et al*., 2000; Ouspenski *et al*., 2000; Lavoie *et al*., 2002; Yu and Koshland, 2003; Abe *et al*., 2011); however, more recent analyses suggest that condensin maintains chromosome architecture rather than mediating compaction itself (Hagstrom *et al*., 2002; Chan *et al*., 2004; Hirota *et al*., 2004; Gerlich *et al*., 2006; Vagnarelli *et al*., 2006; Cuylen *et al*., 2011). Although in many species condensin localizes to the chromosome axis during mitotic cell division, it is also seen to accumulate at specific chromosome regions (Hirano and Mitchison, 1994; Steen *et al*., 2000; Steffensen *et al*., 2001; Beenders *et al*., 2003; Savvidou *et al*., 2005; Cuylen and Haering, 2011). In budding yeast, condensin is concentrated at rDNA throughout mitosis, becoming further enriched onto the rDNA at the start of anaphase, where it has a role in the organization and segregation of the rDNA (Freeman *et al*., 2000; Bhalla *et al*., 2002; Lavoie *et al*., 2002; Yu and Koshland, 2003, 2005; D'Amours *et al*., 2004; Machin *et al*., 2004; Sullivan *et al*., 2004; Wang *et al*., 2004; Wang *et al*., 2005; D'Ambrosio *et al*., 2008a; Nakazawa *et al*., 2008). Specific rDNA localization has not been observed in other species; however, condensin does localize to the nucleolus in human (Cabello *et al*., 2001) and *Arabidopsis thaliana* (Fujimoto *et al*., 2005), suggesting a possible role in rDNA organization. Condensin also localizes to the centromeric DNA in several species, where it has been found to be important for the structural integrity of the centromere (Bachellier-Bassi *et al*., 2008).

One of the most striking phenotypes of condensin mutants is the presence of anaphase bridges between segregating chromosomes in both meiosis and mitosis (Saka *et al*., 1994; Bhat *et al*., 1996; Freeman *et al*., 2000; Lavoie *et al*., 2000, 2002; Ouspenski *et al*., 2000; Steffensen *et al*., 2001; Hagstrom *et al*., 2002; Coelho *et al*., 2003). One explanation that has been suggested for the origin of these bridges is an inability to remove catenations between segregating chromosomes, possibly also involving topoisomerase II (Koshland and Strunnikov, 1996; Hirano, 2000; Holmes and Cozzarelli, 2000; Hirano, 2010).

The role of condensin in meiosis has been investigated in a variety of species, where it has been associated with a number of roles. In budding yeast, which only has the canonical condensin I complex, it is required for chromosome compaction during prophase I and for the normal assembly of the synaptonemal complex (SC; Yu and Koshland, 2003). Studies also show that condensin is required for the removal of cohesin from the chromosome arms in prophase I. Its absence leads to the persistence of recombination-dependent interchromosomal connections (Yu and Koshland, 2005). In *Drosophila*, mutation of the condensin I subunit, CAP-G, did not affect SC formation but led to a delay in SC disassembly and was associated with premature chromosome segregation at metaphase I, leading to aneuploidy (Resnick *et al*., 2009). *Drosophila* condensin II mutants exhibited defects in chromosome territory formation and chromosome individualization (Hartl *et al*., 2008a). Similarly, defects in individualization of meiotic chromosomes have been reported in condensin II depleted mice (Lee *et al*., 2011). Condensin I is also thought to play a role in the structural organization of mouse chromosomes at metaphase I (Viera *et al*., 2007). In the nematode worm, *Caenorhabditis elegans*, three condensin-related complexes have been identified (Csankovszki *et al*., 2009). One of these is a canonical condensin II, which based on the analysis of a mutant in the CAP-D3 subunit homolog HCP-6, indicates a role during both the first and second meiotic divisions in the resolution of cohesin-independent linkages (Chan *et al*., 2004). The dosage compensation complex (DCC) is a specialized condensin-related complex that is involved in X-chromosome silencing. Condensin I contains subunits from both condensin II and DCC. Later studies detected a defect in axial length shortening and CO distribution in condensin II depleted worms (Mets and Meyer, 2009). Condensin II was seen to partially associate with the DNA in pachytene I and to localize fully to the DNA at diplotene and diakinesis, where it persisted on the chromatin throughout the rest of meiosis (Chan *et al*., 2004; Mets and Meyer, 2009).

In *A. thaliana* two partially redundant SMC2 homologs, referred to as AtCAP-E1 and AtCAP-E2, have been identified (Liu *et al*., 2002; Siddiqui *et al*., 2003). Embryo lethality was observed in double mutants and *Atcap-e1:AtCAP-E2/Atcap-e2* plants. In *Atcap-E1/Atcap-e1:AtCAP-e2* plants and antisense RNA knock-down lines a number of developmental phenotypes were observed, including slow growth, fasciation and meristem disorganization. Similar phenotypes were also reported for plants in which the *AtSMC4* gene was disrupted (Siddiqui *et al*., 2006). Although meiosis was not extensively analysed in these studies, cytogenetic analysis of 4′,6-diamidino-2-phenylindole (DAPI)-stained pollen mother cells (PMCs) from *Atcap-E1/Atcap-e1:AtCAP-e2* plants indicated reduced chromatin condensation in some cells and evidence of chromatin bridges at anaphase I. Analysis of *Atcap-D2* and *Atcap-D3* mutants have revealed a role in growth, fertility and chromatin organization (Schubert *et al*., 2013). Homozygous *Atcap-D2* plants are non-viable, indicating that the gene is essential. Heterozygous plants show normal vegetative growth but have reduced fertility. The loss of *AtCAPD-3* is also associated with reduced fertility. A reduction in chromatin density and an increased tendency for centromeric associations was observed in both the *Atcap-d3* mutant and the *Atcap-d2* heterozygote lines (Schubert *et al*., 2013).

In this study we describe a detailed analysis of the role of the condensin complex during meiosis in *A. thaliana*. This reveals that both condensin I and condensin II participate in normal remodelling of the chromosomes during meiosis, although their roles are distinct. Loss or reduction of condensin results in meiotic defects that compromise fertility.

## Results

### Condensin I and condensin II are expressed in Arabidopsis buds

The condensin complex is a pentameric complex that is highly conserved in eukaryotes (Hirano *et al*., 1997). The complex consists of an SMC2/SMC4 backbone and three regulatory subunits that differ between the condensin I and condensin II complexes. These are CAP-H, CAP-D2 and CAP-G for the condensin I complex, and CAP-H2, CAP-D3 and CAP-G for the condensin II complex. Database searches revealed that the Arabidopsis genome encodes subunits of both condensin I and condensin II (Schubert *et al*., 2013). To investigate whether both condensin protein complexes are present, an anti-AtSMC4 antibody (see Experimental procedures) cross-linked to sepharose beads was used to immunoprecipitate AtSMC4-containing complexes from protein extracts prepared from Arabidopsis meiotic buds. Analysis of the protein complexes using mass spectrometry confirmed the presence of components of both condensin complexes (Table S1).

### Condensin associates with the chromosomes throughout meiosis

**Figure 1:**
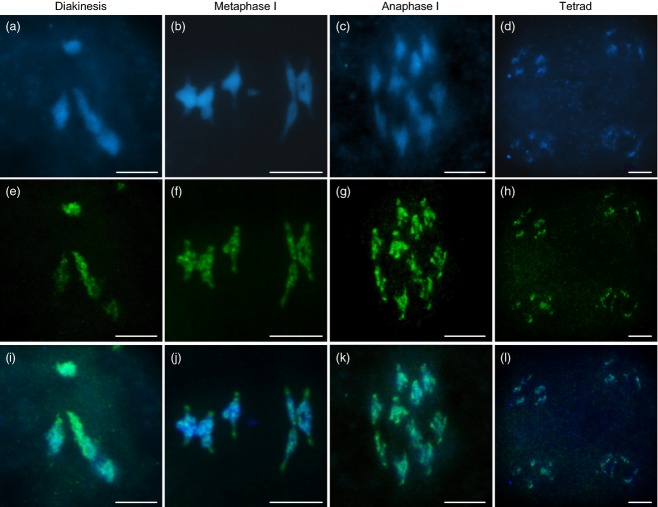
Immunolocalization of AtSMC4 on wild-type pollen mother cells (PMCs); (a–d) DAPI (blue); (e–h) AtSMC4 (green); (i–l) DAPI and AtSMC4 merged. Scale bar: 5 μm.

**Figure 2:**
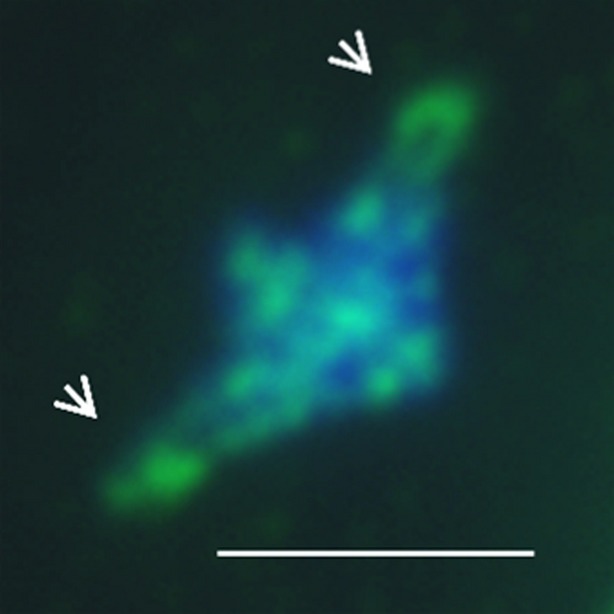
Immunolocalization of AtSMC4 on wild-type at metaphase I; DAPI (blue) and AtSMC4 (green), merged. Arrowheads indicate centromere regions with more intense AtSMC4 staining. Scale bar: 5 μm.

Immunolocalization using an anti-AtSMC4 antibody was used to investigate the distribution of condensin in chromosome-spread preparations from PMCs at different meiotic stages (Figure1). Throughout most of prophase I the complex was not detectable, but as the chromosomes condensed and individualized at the end of prophase I, and progressed through the meiotic divisions (Figure1a–c), an AtSMC4 signal became apparent (Figure1e–g,i–k). Inspection of the chromosomes at metaphase I and anaphase I suggested that the complex localized throughout the chromosomes; however, the signal strength was variable, with small patches of increased intensity. In particular, it appeared that the signal was slightly more intense at the centromeric regions (Figure2). Staining remained throughout the chromosomes until tetrad formation (Figure1d,h,l). No staining was observed using pre-immune serum as a control (Figure S1).

### Fertility is reduced in *Atsmc4/SMC4* heterozygotes

Previous studies have shown that disruption of either the SMC2 or the SMC4 subunit of condensin results in embryo lethality in *A. thaliana* (Siddiqui *et al*., 2003, 2006); however, lines in which a threshold level of the condensin complex was expressed survived, albeit with a variety of developmental defects (Siddiqui *et al*., 2003, 2006). To investigate the meiotic role of condensin in more detail we began by analysing the previously described *Atsmc4* allele (At5g48600, Sail_86_D2) that contains a T-DNA insertion in the seventh intron (Figure S2). In accordance with the earlier study we failed to identify homozygous lines. Genotyping of 124 plants indicated that 105 were wild-type and 19 were *AtSMC4/Atsmc4* heterozygotes, representing a significant deviation from a Mendelian segregation (*P* < 0.001). The heterozygous plants had a normal vegetative phenotype, but produced shorter siliques with fewer seed than wild-type controls.

**Figure 3:**
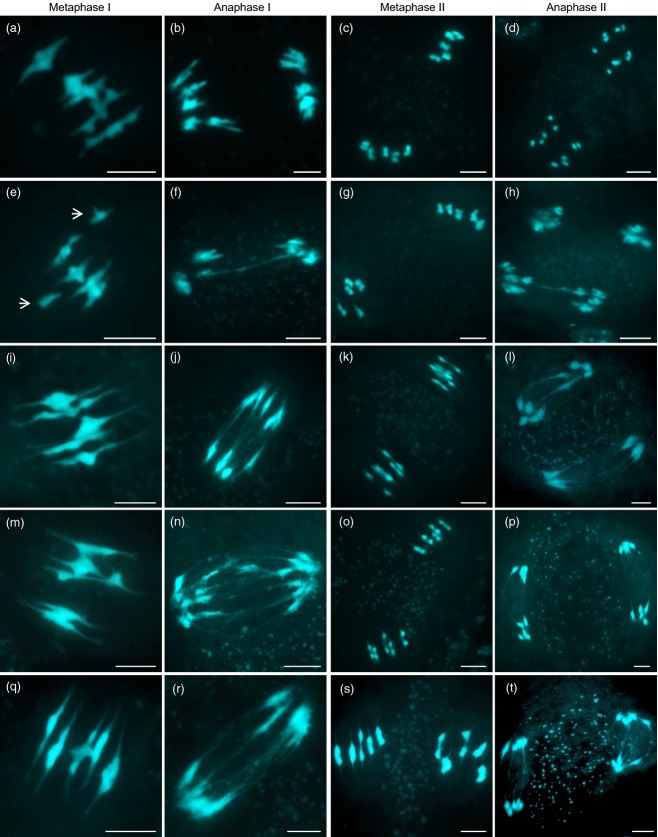
DAPI-stained chromosome spreads of AtSMC4-depleted pollen mother cells (PMCs) at the first and second meiotic divisions: (a–d) wild-type control; (e–h) *AtSMC4*/*Atsmc4*; (i–l) *AtSMC4*^*RNAi-1*^; (m–p) *AtSMC4*^*RNAi-2*^; (q–t) *AtCAP-D*2^*RNAi*^. Arrows in (e) indicate univalent chromosomes. Scale bar: 5 μm.

To determine whether errors during meiosis were likely to be a factor in the reduced fertility of the *AtSMC4/Atsmc4* plants, a cytogenetic analysis of DAPI-stained chromosome spreads from PMCs was conducted. Inspection of nuclei from G2 through to the end of prophase I showed no discernible difference from corresponding wild-type controls, with the homologous chromosomes pairing and synapsing as normal (Figure S3a,c). At metaphase I five condensed bivalent chromosomes were observed in wild-type PMCs (Figure3a), and in the majority of *AtSMC4/Atsmc4* PMCs (18/20 cells), but in two of the sample a pair of univalent chromosomes was observed (Figure3e). This was not observed in the wild-type and we have not previously observed this in wild-type plants. Both anaphase I and anaphase II were characterized by the frequent presence of connections between some segregating chromosomes (Figure3f,h). These connections were absent in the corresponding wild-type cells (Figure3b,d).

### Reduction of AtSMC4 during meiosis affects chromosome condensation and chiasma frequency

**Figure 4:**
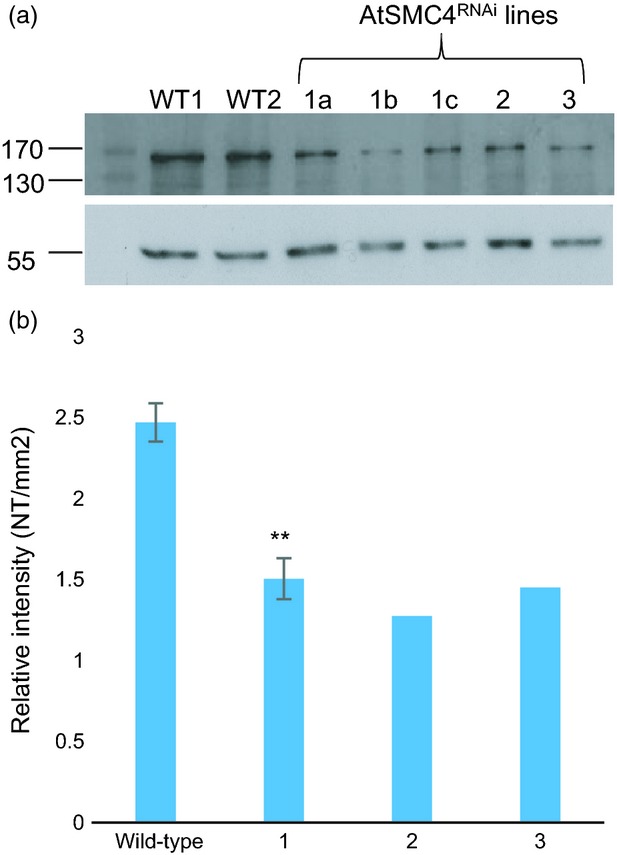
(a) Western blot showing relative intensities of AtSMC4 protein in anthers from wild-type (WT) and *AtSMC4*^*RNAi*^ plants (above) with tubulin loading control (below): 1a, *AtSMC4*^*RNAi-1*^ plant A; 1b, *AtSMC4*^*RNAi-1*^ plant B; 1c, *AtSMC4*^*RNAi-1*^ plant C; 2, *AtSMC4*^*RNAi-2*^; 3, *AtSMC4*^*RNAi-3*^.(b) Relative intensities of the AtSMC4 protein bands adjusted for tubulin loading from the gel shown above: wild-type, average of wild-type samples WT1 and WT2; 1, average intensity of *AtSMC4*^*RNAi-1*^ plants A–C (lanes 1–3 above), **P < 0.05, error bars indicate the standard error of the mean; 2, *AtSMC4*^*RNAi-2*^; 3, *AtSMC4*^*RNAi-3*^.

To gain more insight into the meiotic role of condensin we sought to specifically reduce the expression of *AtSMC4* during this process using RNA interference (RNAi). A 387-bp segment of *AtSMC4* (residues 1633–2020) was used as the basis of an RNAi construct (see Experimental procedures) and cloned into the pPF408 binary vector (Siaud *et al*., 2004), thereby bringing its expression under the control of the *AtDMC1* meiotic promoter (Klimyuk and Jones, 1997; Higgins *et al*., 2005). After initial selection by BASTA resistance, a total of 14 plant lines were identified that all shared the same meiotic phenotype as described below. Following screening of independent transgenic lines from the T_2_ generation exhibiting a 3:1 segregation ratio, three lines with reduced fertility were selected. Initial attempts using RT-PCR to confirm that *AtSMC4* expression was reduced in these lines proved inconclusive, because we were obliged to conduct the analysis using anther tissue, which contains vegetative cells as well as meiotic cells that could not be readily isolated. As an alternative, we found western blotting of anther protein extracts to be more robust. This indicated that each of the three lines exhibited a moderate reduction in the level of AtSMC4 protein present in their anthers (Figure4). Quantification of the AtSMC4 signal indicated that the level of protein was reduced to 50–60% of the wild-type level. Nevertheless, this probably underestimates the degree of AtSMC4 depletion in the PMCs for the reason mentioned above. During the course of the study it became apparent that the transgene in one of the lines was silenced, hence the remaining two lines, referred to as *AtSMC4*^*RNAi-1*^ and *AtSMC4*^*RNAi-2*^, were retained for further analysis. Both lines exhibited a significant reduction in fertility. *AtSMC4*^*RNAi-1*^ had a 29% reduction in seed set compared with the wild-type, and *AtSMC4*^*RNAi-2*^ had a 51% reduction. Pollen viability, as determined by Alexander staining (Alexander, 1969), revealed a significant reduction in the ratio of viable to non-viable pollen between wild-type and *AtSMC4*^*RNAi*^ plants [wild-type, viable pollen:non-viable pollen, 138.6:1 (*n* = 4927); *AtSMC4*^*RNAi-1*^, viable pollen:non-viable pollen, 89:1 (*n* = 5261; *P* < 0.005); *AtSMC4*^*RNAi-2*^, viable pollen:non-viable pollen, 28:1 (*n* = 1546; *P* < 0.005)].

Cytological analysis of DAPI-stained chromosome-spread preparations from *AtSMC4*^*RNAi*^*-*^*1*^ PMCs revealed no obvious differences compared with wild-type during prophase I (Figure S3a,e). Consistent with this, immunolocalization of the chromosome axis component, AtASY1, and the synaptonemal transverse filament protein, AtZYP1, on chromosome-spread preparations of *AtSMC4*^*RNAi-1*^ appeared identical to those from wild-type and *AtSMC4/Atsmc4* PMCs (Figure S3b,d,f). The chromosome axes were elaborated at leptotene, with short stretches of AtZYP1 appearing along the chromosomes at early zygotene and fully polymerizing along the synapsed homologs at pachytene. To determine whether there was any overall effect on the chromosome axes, the mean axis length was determined at pachytene stage for *AtSMC4*^*RNAi-1*^ relative to the wild-type, but no significant difference was observed (*P* = 0.26).

Despite prophase I appearing normal, the cytological analysis of *AtSMC4*^*RNAi-1*^ and *AtSMC4*^*RNAi-2*^ revealed substantial defects at both the first and second meiotic divisions (Figure3i–p). In wild-type metaphase I, chromosomes appear as highly condensed structures (Figure3a); however, in *AtSMC4*^*RNAi-1*^ and *AtSMC4*^*RNAi-2*^ the metaphase I chromosomes appeared more stretched than normal (Figure3i,m). Immunolocalization with the anti-SMC4 antibody revealed that the distribution of the protein at metaphase I was substantially reduced relative to the wild-type (Figure S4, compare panels b and c with e and f). At anaphase I, when the chromosomes segregated to the opposite poles, multiple thin threads of chromatin were observed between the segregating chromosomes in *AtSMC4*^*RNAi-1*^ and *AtSMC4*^*RNAi-2*^ PMCs, which were not present in the wild-type (Figure3b,j,n). At metaphase II the wild-type chromosomes were condensed into discrete units, whereas the *AtSMC4*^*RNAi*^ chromosomes appeared elongated and misshapen (Figure3c,k,o). Thin ‘curtains’ of chromatin, reminiscent of those observed in anaphase I, were seen between the chromosomes at anaphase II that were not present in the wild-type (Figure3d,l,p). These phenotypic characteristics were not observed in control plants transformed with the empty pPF408 vector, indicating that they arose as a consequence of reduced levels of AtSMC4.

As our analysis of the *AtSMC4/Atsmc4* T-DNA line suggested a possible effect on chiasma formation, we determined the chiasma frequency in the *AtSMC4*^*RNAi-1*^ and *AtSMC4*^*RNAi-2*^ lines. The chiasma frequency was scored at metaphase I in chromosome-spread preparations labelled using fluorescence *in situ* hybridization (FISH) with *45S* and *5S* ribosomal (rDNA) to distinguish the individual chromosomes (Sanchez-Moran *et al*., 2001). A slight reduction in the mean chiasma frequency for both lines was detected, from 9.05 chiasma per cell for the wild-type (*n* = 20) to 8.44 in *AtSMC4*^*RNAi-1*^ (*P* = 0.0531, *n* = 32) and 8.15 in *AtSMC4*^*RNAi-2*^ (*P* = 0.007, *n* = 25).

### Organization of the centromeric DNA and rDNA is perturbed in the *AtSMC4*^*RNAi*^ lines

**Figure 5:**
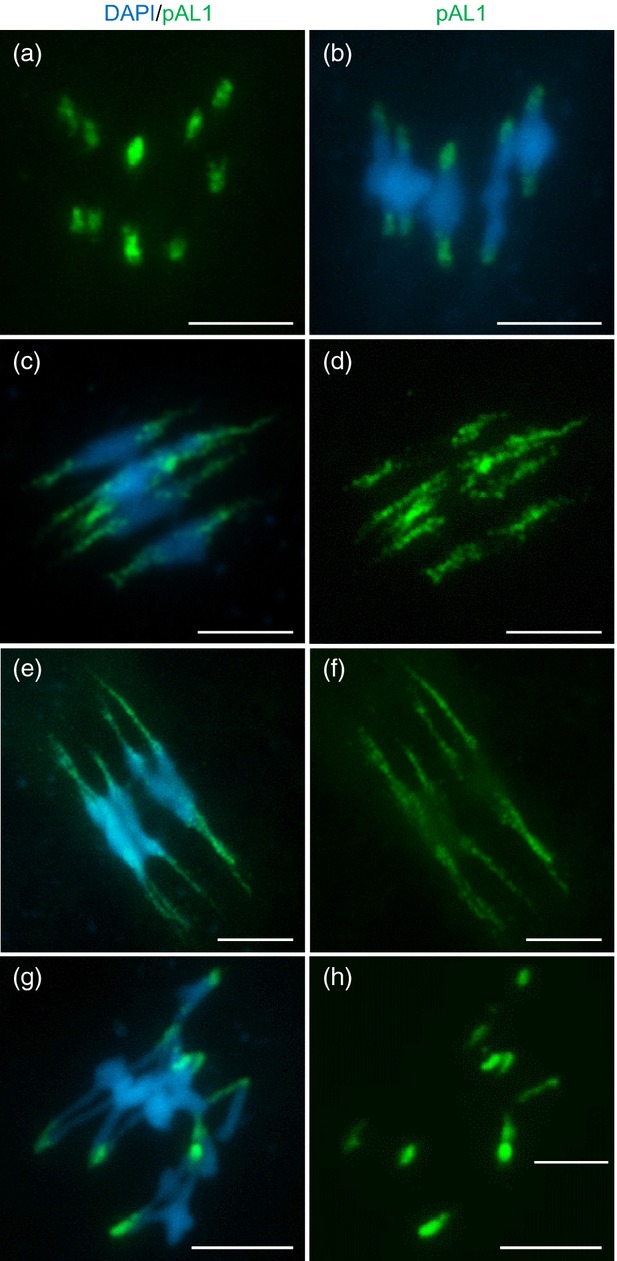
Fluorescence *in situ* hybridization using pericentromeric-specific probe pAL1 (green) on metaphase-I cells of wild-type and condensin-depleted plants: (a, b) wild-type; (c, d) *AtSMC4*^*RNAi*^; (e, f) *AtCAP-D2*^*RNAi*^; (g, h) *Atcap-d3*. Scale bar: 5 μm.

Both the centromeres and rDNA are comprised of repetitive DNA sequences, and condensin has been implicated in the organization of both these regions (Freeman *et al*., 2000; Bhalla *et al*., 2002; Lavoie *et al*., 2002; D'Amours *et al*., 2004; Machin *et al*., 2004; Sullivan *et al*., 2004; Wang *et al*., 2004, 2005; Oliveira *et al*., 2005; Savvidou *et al*., 2005; Gerlich *et al*., 2006; Yong-Gonzalez *et al*., 2007; Nakazawa *et al*., 2008; Ribeiro *et al*., 2009; Samoshkin *et al*., 2009). To investigate whether Arabidopsis condensin is involved in centromeric organization of the meiotic chromosomes, a FISH analysis was conducted using the pericentromeric probe pAL1 (Martinez-Zapater *et al*., 1986) on chromosome-spread preparations from wild-type and *AtSMC4*^*RNAi*^ PMCs. At prophase I the centromeric signal appeared discrete and relatively condensed in both cases (Figure S5); however, at metaphase I, whereas the wild-type signals remained compact (Figure5a,b), those in the *AtSMC4*^*RNAi*^ lines were markedly stretched (Figure5c,d).

**Figure 6:**
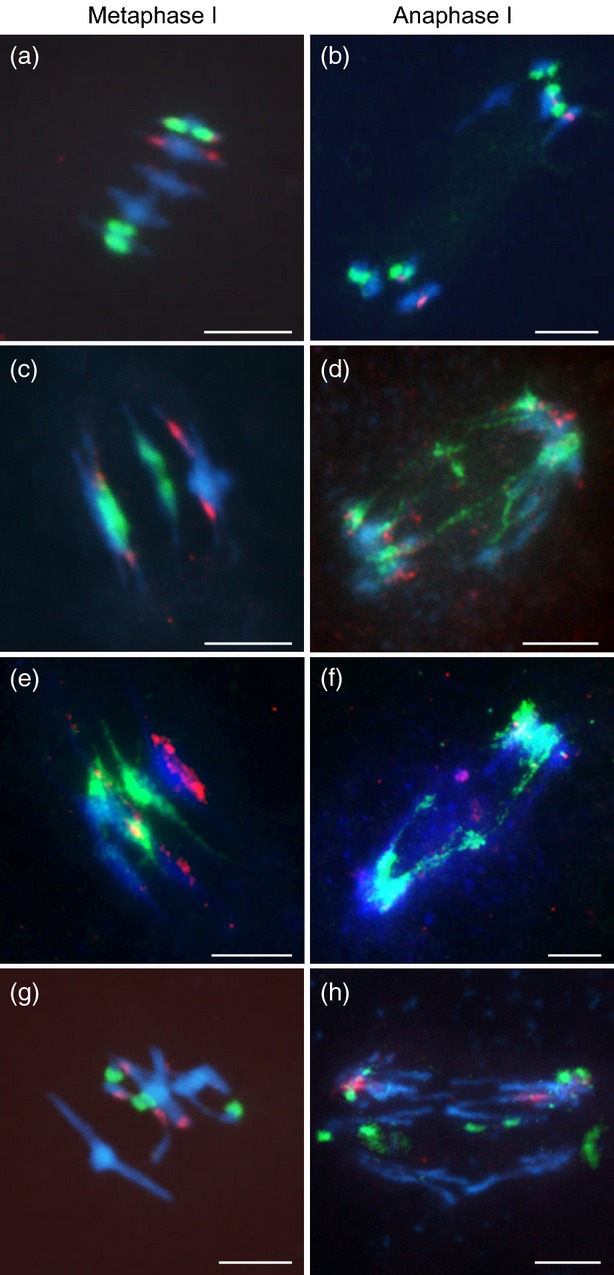
Fluorescence *in situ* hybridization using rDNA-specific probes *45S* (green) and *5S* (red) on metaphase-I and anaphase-I pollen mother cells (PMCs) of wild-type and condensin-depleted plants: (a, b) wild-type; (c, d) *AtSMC4*^*RNAi*^; (e, f) *AtCAP-D2*^*RNAi*^; (g, h) *Atcap-d3*. Scale bar: 5 μm.

To investigate the integrity of the rDNA during meiosis in the *AtSMC4*^*RNAi*^ lines, FISH analysis was conducted using the *45S* and *5S* rDNA probes Sanchez-Moran *et al*., 2001). There were no obvious differences between the wild type and *AtSMC4*^*RNAi*^ at prophase I, but at metaphase I the signals appeared enlarged in *AtSMC4*^*RNAi*^ (Figure6a–d). To determine if this resulted from a reduction in compaction of the whole chromosome or if there was a specific effect on the rDNA, the ratio of the rDNA signal to overall chromosome length was investigated. The *5S* signal on chromosome 5 and the *45S* signal on chromosome 4 were analysed. This indicated no significant defect in the *45S* condensation in the RNAi lines relative to the wild-type (wild-type ratio 0.363, *n* = 20; *AtSMC4*^*RNAi-1*^ ratio 0.415, *P* = 0.09, *n* = 20; *AtSMC4*^*RNAi-2*^ ratio 0.333, *P* = 0.37, *n* = 8), whereas there was a significant difference for the *5S* region (wild-type ratio 0.202, *n* = 20; *AtSMC4*^*RNAi-1*^ ratio 0.272, *P* = 0.001, *n* = 27; *AtSMC4*^*RNAi-2*^ ratio 0.267, *P* = 0.015, *n* = 8). Metaphase II appeared to be similar. The defect becomes most obvious at anaphase I and anaphase II, when the *45S* signal spans the gap between the segregating chromosomes. In a few nuclei the *5S* rDNA signal was also observed to span the region between the segregating chromosomes, suggesting that despite no obvious effect at metaphase I, it too had a condensation defect. Nevertheless, the rDNA signals did not account for all the lagging chromatin at anaphase I, as chromatin threads were seen between all five pairs of segregating chromosomes (Figure6d).

### Distinct roles for the condensin complexes

As reducing AtSMC4 expression compromises both condensin subunits, we sought to determine the role of the individual complexes during meiosis. DNA sequence analysis confirmed that Sail_826_B06 contained a single T-DNA insertion in the first exon of the condensin II-specific subunit *AtCAP-D3* at residue 1089. As a result, a transcript corresponding to the gene was absent from both vegetative and reproductive tissues (Figure S6). This suggests that the AtCAP-D3 protein would not be expressed, and thus a functional condensin II complex would be absent. The *Atcap-d3* plants exhibited a distinct dwarfed phenotype and possessed small rosette leaves, compared with the wild-type (Figure S7). They also had a significant reduction in seed-set, to around 40% of that in the wild type. Moreover, only 63.75% (*n* = 400) of this seed was viable, compared with 100% of the wild-type seed (*n* = 400).

**Figure 7:**
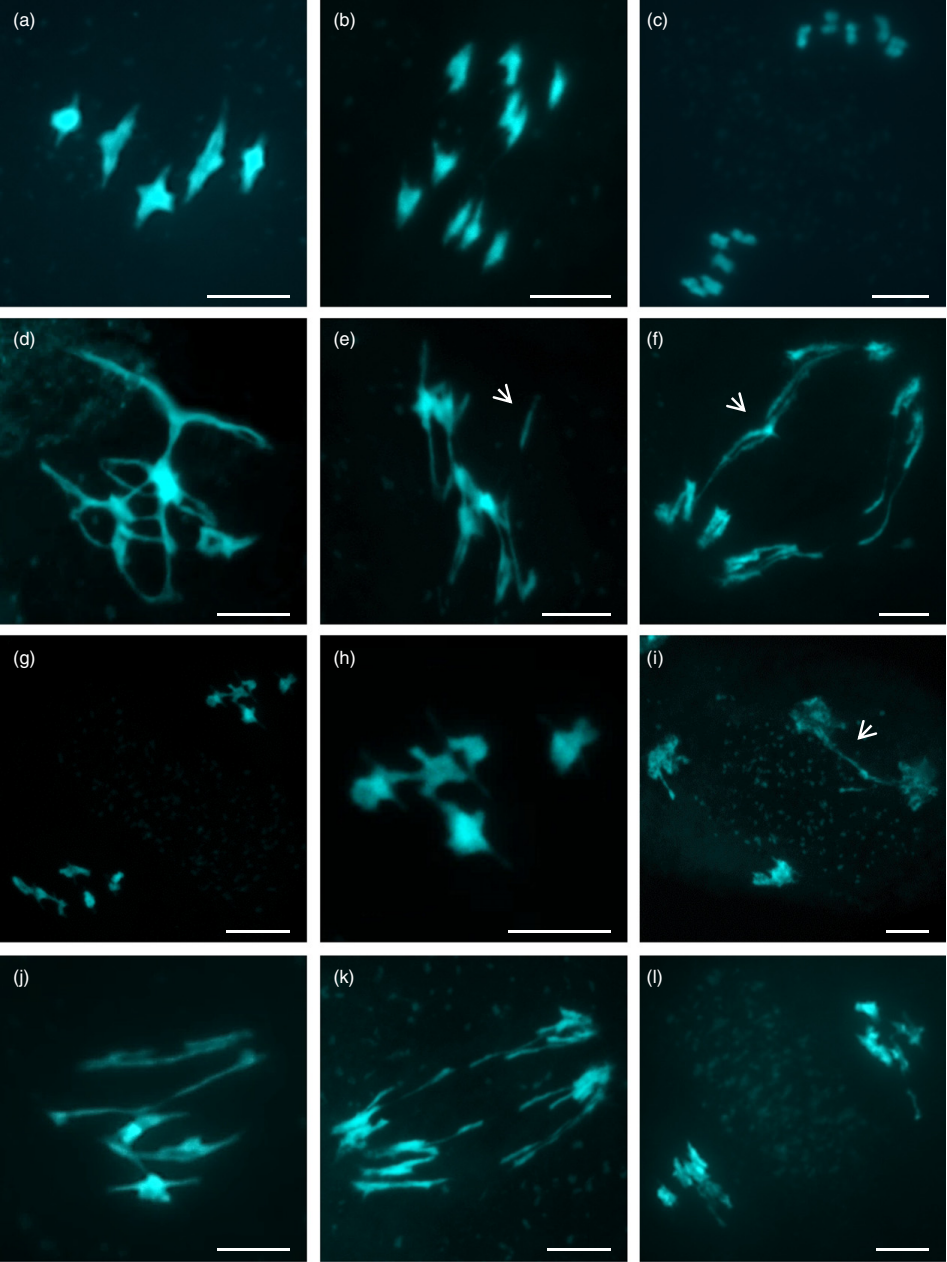
DAPI-stained chromosome spreads of AtCAP-D3-depleted pollen mother cells (PMCs): (a–c) wild-type control; (d–i) *Atcap-d3*; (j–l) *AtCAP-D3*^*RNAi*^; (a, d, e, j) metaphase I; (b, f, k) anaphase I; (c, g, h, l) metaphase II (panel h is a magnification of panel g); (i) tetrad. Scale bar: 5 μm. Arrows indicate a chromosome fragment (e) and chromosome bridges (f, i).

To determine whether the reduced fertility was associated with defects in meiosis, a cytological analysis of chromosome-spread preparations from *Atcap-d3* PMCs was conducted. This revealed various abnormalities. Prophase I was apparently normal, with chromosomes achieving complete synapsis at pachytene. This result was confirmed by immunolocalization of AtASY1 and AtZYP1 in prophase-I PMCs of *Atcap-d3* and wild-type plants, which revealed no differences (Figure S3b,h). At metaphase I the *Atcap-d3* chromosomes appeared stretched, with multiple chromosome associations involving all five bivalents observed in most cells (31/33; Figure7d,e). Chromosome fragments were observed in a few cells (2/33), which may have arisen from problems in resolving the chromosome associations or from unrepaired DNA double-strand breaks (DSBs) (Figure7e). At anaphase I the chromosomes did not appear to migrate to the poles in unison. Instead, trailing chromosomes were observed with, in some cases (4/13), connections between the segregating chromosomes (Figure7f). At metaphase II the chromosomes appeared less condensed than in the wild-type, with connections between the chromatids (Figure7g,h). Similar to anaphase I, stretched trailing chromosomes were observed at anaphase II. In tetrad cells the chromatin appeared as a rather fuzzy mass instead of the comparatively organized discrete chromosomes observed in the wild-type. In one instance an *Atcap-d3* tetrad with a chromatin connection between the separated chromatids was observed (Figure7i).

To confirm the *Atcap-d3* phenotype and to investigate the contribution of condensin I we sought to knock-down expression of *AtCAP-D3* and *AtCAP-D2* using RNAi. Given the problem with using RT-PCR to estimate PMC-specific gene knock-down encountered with *AtSMC4*, we decided to screen on the basis of reduced fertility and cytological analysis. Our rationale was that an *AtCAP-D3* RNAi knock-down should confirm the *Atcap-d3* mutant phenotype, whereas an *AtCAP-D2* knock-down might reveal phenotypic characteristics that were absent in lines lacking AtCAP-D3 but observed when both subunits were compromised, as in the lines where AtSMC4 expression was reduced.

A 524-bp segment (bases 2109–2633) of *AtCAP-D3* was used as the basis for the RNAi construct in pPF408. Following an initial screen for reduced fertility, 10 lines were selected for cytological analysis. The fertility of these lines ranged between approximately 25 and 96% of the wild-type level. Cytological analysis of meiotic chromosome spreads from these lines revealed no detectable defects in prophase I; however, the same abnormalities in chromosome organization and segregation that had been observed in *Atcap-d3* were present from metaphase I through to the tetrad stage. Unsurprisingly, there was some variation in the severity of the defects, which was consistent with the degree by which overall fertility was reduced in each case, with those having a seed-set comparable with *Atcap-d3* exhibiting a cytological phenotype that was indistinguishable from the T-DNA insertion line (Figure7j–l).

In the case of *AtCAP-D2,* a 702-bp region (bases 1069–1771) was used to make a knock-down construct in pPF408. After recovery of the transformed lines, 12 lines with reduced fertility, spanning a range from approximately 11 to 98% of that in the wild-type, were selected for cytological analysis. Inspection of chromosome-spread preparations revealed that prophase I was apparently normal (Figure S3i). Chromosome spreads of the remaining meiotic stages revealed that at metaphase I the chromosomes were rather elongated compared with those in the wild-type, and at both subsequent divisions chromatin connections between the segregating chromosomes were present (Figure3q–t). The lines exhibited a range in severity that more or less correlated with the range of observed fertility defects, and presumably with the degree of *AtCAP-D2* knock-down.

To pursue the analysis further, FISH using the *45S* and *5S* rDNA and centromeric probes was carried out on the *AtCAP-D2*^*RNAi*^ and *Atcap-d3* lines. In essence, the presumed reduction in *AtCAP-D2* appeared to lead to defects reminiscent of those observed when *AtSMC4* expression was reduced. The centromeric DNA appeared normal during prophase I but at metaphase I had a ‘stretched’ appearance (Figure5e,f). Analysis of the *45S* and *5S* rDNA showed that the signals at metaphase I and metaphase II were diffuse, spreading out along the chromosomes carrying the rDNA. If anything, this effect was more pronounced than in the *AtSMC4*^*RNAi*^ lines, but this could reflect variation in the relative reductions in gene expression. At anaphase I, stretched trailing strands of rDNA were visible between the segregating chromosomes, as observed in the *AtSMC4*^*RNAi*^ lines (Figure6f). When FISH analysis of the *Atcap-d3* line was carried out, a different picture emerged. Although, the chromosomes exhibited extensive interconnections, as far as could be judged, the centromeric DNA (Figure5g,h) and the rDNA (Figure6g,h) signals remained relatively condensed. Thus, it would appear that normal organization of the centromeric repeats and rDNA on Arabidopsis meiotic chromosomes requires the condensin I complex, but remains largely unaffected by a mutation to one of the condensin II subunits. Nevertheless, it seems that overall chromosome organization during meiosis requires both condensin complexes.

## Discussion

The condensins are members of the evolutionarily conserved SMC family of proteins. Although they have been studied in a wide range of species, their analysis in plants has been relatively limited. Previous studies in Arabidopsis have shown that the total loss of condensin is lethal. Thus, to investigate the role that the complex plays during meiosis, we examined plants in which condensin expression was compromised but not entirely absent. To achieve this it was necessary to use RNAi lines in which reduced expression of components of the condensin complexes was restricted to meiotic cells. A consequence of this was that direct measurement of the reduction in expression of the target gene was not feasible; however, direct comparison of the *Atcap-d3* T-DNA knock-out line and *AtCAP-D3*^*RNAi*^ lines with similar levels of fertility indicated that seed-set could be used as a proxy to identify lines where expression of the target gene was substantially reduced. Despite this limitation these studies provide substantial insight into the role of condensin during meiosis in Arabidopsis. A range of defects have been identified that impact on the meiotic pathway that, in turn, negatively impacts on pollen viability and fertility.

### Loss or depletion of condensin affects meiotic chromosome structure

Examination of metaphase I bivalents revealed that depletion of condensin I and condensin II, either independently or together, resulted in elongated bivalents at metaphase I. Nevertheless, it was clear that a significant level of chromosome condensation was still achieved. Although this may represent a hypomorphic state arising from residual condensin in the lines examined, it could suggest that condensin in Arabidopsis, unlike in Xenopus and yeast (Saka *et al*., 1994; Strunnikov *et al*., 1995; Lieb *et al*., 1996; Hirano *et al*., 1997; Sutani *et al*., 1999; Freeman *et al*., 2000; Ouspenski *et al*., 2000; Lavoie *et al*., 2002; Yu and Koshland, 2003), is not essential for the overall condensation of the chromosomes. Alternatively, it may indicate a role for maintaining chromosome condensation. In support of this, AtSMC4 localization to the chromatin was first observed in wild-type PMCs at late diakinesis, when the chromosomes had already largely condensed. A similar conclusion has been suggested for condensin in *C. elegans* (Hagstrom *et al*., 2002; Chan *et al*., 2004), chicken (Hudson *et al*., 2003), human (Ono *et al*., 2003; Hirota *et al*., 2004; Gerlich *et al*., 2006) and *Drosophila* (Savvidou *et al*., 2005).

### Distinct phenotypes are associated with the depletion of condensin I and condensin II

Our data indicate that the two condensin complexes are functionally non-redundant. This is consistent with results from other species where the two complexes have been depleted separately (Ono *et al*., 2003; Gerlich *et al*., 2006; Shintomi and Hirano, 2011). Selective reduction of components of the Arabidopsis condensin I and condensin II complexes through mutation or RNAi has revealed a role for the former in condensation of the centromeric and *45S* rDNA. Condensin has been shown to have a role in the organization of the centromeres in many species (Wignall *et al*. 2003; Ono *et al*., 2004; Jager *et al*., 2005; Oliveira *et al*., 2005; Savvidou *et al*., 2005; Yong-Gonzalez *et al*., 2007; Samoshkin *et al*., 2009; Bernad *et al*., 2011). At metaphase I and metaphase II in the *AtSMC4*^*RNAi*^ and *AtCAP-D2*^*RNAi*^ lines, the centromeres appeared elongated compared with the wild-type. This phenotype was not observed in lines lacking AtCAP-D3. This implies that condensin I, but not condensin II, is required to maintain centromere structure in Arabidopsis, and that its loss may contribute to the elongated nature of the metaphase-I chromosomes. The pulling force of the spindle when the centromeres are aligned along the metaphase plate may be responsible for the distortion of the centromeric DNA. This may be caused either from a loss of centromere stiffness after normal spindle attachment, as is seen in other systems (Oliveira *et al*., 2005; Savvidou *et al*., 2005; Gerlich *et al*., 2006; Ribeiro *et al*., 2009), or from abnormal merotelic attachment, as shown in some studies (Stear and Roth, 2002; Samoshkin *et al*., 2009; Tada *et al*., 2011). These stretched centromeres are, however, still able to segregate the chromosomes to the opposite poles of the cells, although it is likely that there is a delay in this process because a high number of anaphase I and II cells are detected in condensin I-depleted lines, compared with the wild-type.

A similar separation of function between the condensin complexes was also observed in relation to condensation of the *45S* rDNA. The rDNA comprises a large quantity of repetitive DNA and it is therefore important to maintain the structural integrity of this unit during cell division and homologous recombination. A role for condensin in relation to rDNA organization might therefore be anticipated and, consistent with this, condensin has previously been implicated in rDNA maintenance in budding yeast (Freeman *et al*., 2000; Bhalla *et al*., 2002; Lavoie *et al*., 2002; D'Amours *et al*., 2004; Lavoie *et al*., 2004; Machin *et al*., 2004; Sullivan *et al*., 2004; Wang *et al*., 2004, 2005; Tsang *et al*. 2007; D'Ambrosio *et al*., 2008a; Nakazawa *et al*., 2008), where it may be required to prevent recombination occurring between rDNA repeats (Bhalla *et al*., 2002), or to help prevent or remove catenations in the rDNA regions (D'Ambrosio *et al*., 2008a). Our data reveal a similar role for condensin I during plant meiosis. The *45S* rDNA in the *AtSMC4*^*RNAi*^ and *AtCAP-D2*^*RNAi*^ lines did not appear as well condensed at metaphase I or at metaphase II, compared with the wild-type. The rDNA regions were also seen to span the gap between segregating chromosomes at meiotic anaphase I and anaphase II in both lines. These phenotypes were not observed in plants lacking condensin II, which appeared normal in relation to the organization of the *45S* rDNA. Thus our data reveal that condensin is required for the organization of the rDNA in a higher eukaryote, similar to that previously reported in budding yeast.

Analysis of the *Atcap-d3* mutant and several *AtCAP-D3* knock-down lines revealed two defects likely to be associated with the loss of condensin II. Condensin has been implicated in the prevention and removal of connections between chromosomes working in conjunction with topoisomerase II (Chan *et al*., 2004). It is conceivable that a similar process occurs in Arabidopsis, which is compromised in the *Atcap-d3* mutant, resulting in multiple connections between the bivalents at metaphase I. The chromosome fragments observed in a few cells at anaphase I may have arisen when these connections were pulled apart at anaphase I. Nevertheless, many of the metaphase I connections observed appeared to be resolved before anaphase I, as only a low frequency of connections was seen between segregating chromosomes at anaphase I. Although lagging threads of chromatin were observed at anaphase I and anaphase II in the condensin I-depleted lines, in the *Atcap-d3* and *AtCAP-D3*^*RNAi*^ lines thicker threads of trailing chromatin were observed at the corresponding stages. It also appeared that the chromosomes lost some of their overall structural integrity. These phenotypes were not particularly obvious in the plants where AtSMC4 expression was reduced, which presumably retain some functional condensin II, suggesting they are sensitive to the level of complex present in the cell.

### A role for condensin during meiotic prophase I?

In addition to the roles of condensin discussed thus far, in some species evidence of an earlier role during meiotic prophase I have also been reported. In *C. elegans* and budding yeast, studies indicate that condensin is required for correct prophase I axis length condensation (Yu and Koshland, 2003, 2005; Tsai *et al*., 2008). A role in synaptonemal complex (SC) assembly has also been reported in budding yeast, where Ycs4 co-localizes with the SC transverse filament protein, Zip1, forming semi-continuous foci (Yu and Koshland, 2003, 2005).

In this study we were unable to show any direct defects in the structure of pachytene chromosomes in any of the condensin-depleted plants analysed. Although this could suggest that in Arabidopsis condensin does not have the same role in prophase I as is seen in other species, we cannot exclude the possibility that the residual condensin in the lines analysed was sufficient to maintain normal or near normal chromosome axes during prophase I. Likewise, we cannot rule out the possibility that the failure to detect condensin by immunolocalization during prophase I in the wild-type simply resulted from the procedure lacking sufficient sensitivity to detect the complexes on the chromosomes at this stage. Another possibility is that the AtSMC4 epitopes are masked by other chromosome-associated components, such as chromosome axis proteins during prophase I. Nevertheless, our data do suggest a role prior to the meiotic divisions. The finding that the depletion of condensin was associated with a slight, yet significant, reduction in chiasma frequency and the occasional presence of univalents at metaphase I suggests some impact on meiotic recombination. As recombination occurs during meiotic prophase I, this would imply a role for condensin during this stage despite our inability to detect it by immunolocalization. A role for condensin during meiotic recombination has previously been reported for budding yeast, where it is implicated in the resolution of recombination-dependent linkages (Yu and Koshland, 2003).

Together these findings provide strong evidence that condensin plays an important and complex role in the structural organization of the chromosomes during meiosis in *A. thaliana*.

## Experimental procedures

### Plant cultivation

*Arabidopsis thaliana* ecotype Columbia 0 (Col-0) was used in this study for wild-type analysis. T-DNA insertion lines were obtained from the European Arabidopsis Stock Centre (uNASC, http://arabidopsis.info). Plants were grown, material was harvested and nucleic acid extractions were performed as previously described by Higgins *et al*. (2004).

### Semi-quantitative RT-PCR analysis of *AtCAP-D3* transcripts

RT-PCR was carried out as previously described (Higgins *et al*., 2004). The primers used were: D3 RTPCRf, 5′-CCTGAGAAGGCCGAGCCGCGTGG-3′; D3 RTPCRr, 5′-CATATTCTGAATGCCTCGGAAATAGC-3′; GAPD-N, 5′-CTTGAAGGGTGGTGCCAAGAAGG-3′; GAPD-C, 5′-CCTGTTGTCGCCAACGAAGTCAG-3′.

### Production of RNAi lines

A 387-bp region of *AtSMC4* cDNA (between bases 1633 and 2020), a 702-bp region of *AtCAP-D2* (between bases 1069 and 1771) and a 524-bp region of *AtCAP-D3* cDNA (between bases 2109 and 2633) were selected to make RNAi constructs. Sequences were used in a BLAST search to check for similarity to other sequences in order to reduce the chances of ‘off-target’ effects caused by the RNAi. PCR fragments for cloning were amplified with the following primers from wild-type Col-0 bud cDNA: SMC4RNAi_*Eco*R1, 5′-CCGAATTCCTTTGCCACAACAGTGTTTC-3′; SMC4RNAi_ *Xho*1, 5′-CGCTCGAGAAGAGTCAGAATGAGG-3′; SMC4RNAi_ *Hind*III, 5′-GCAAGCTTTGCCACAACAGTGTTT-3′; SMC4RNAi_ *Bam*HI, 5′-CGGGATCCGAGAAGAGTCAGAATGAGG-3′; CAPD2_*Bam*HI_f1, 5′-GGGATCCGAGGGAGATATGAGTTC-3′; CAPD2_*Xho*I_f2, 5′-GCTCGAGGGAGATATGAGTTCC-3′; CAPD2_*Hind*III_r3, 5′-GAAGCTTCTGACCGTCAATTTGG-3′; CAPD2_*Eco*R1, 5′-GGAATTCTGCACCGTCAATTTGG-3′; CAPD3_*Bam*H1_f1, 5′-GGGATCCGAGCCTGCTGCAGATCGGA-3′; CAPD3_*Xho*I_f2, 5′-GCTCGAGCCTGCTGCAGATCTGGC-3′; CAPD3_*Hind*III_r3, 5′-GAAGCTTCCCGTCAGCCAAACACATC-3′; CAPD3_*Eco*RI_r4, 5′-GGAATTCCCGTCAGCCAAACACATC-3′.

Polymerase chain reaction (PCR) fragments were cloned into pHANNIBAL (Wesley *et al*. 2001) in sense and antisense orientations. The two inverted sequences, separated by an intron, and the terminator were then subcloned into pPF408, downstream of the AtDMC1 promoter (Klimyuk and Jones, 1997; Higgins *et al*., 2005). The construct was transformed into *Agrobacterium tumefaciens* (GV3101) then subsequently transformed into wild-type Col-0 *A. thaliana* plants using the floral-dip method (as described in Higgins *et al*., 2004). To select transformants, plants were either grown on MS plates containing 25 μg ml^−1^ BASTA (d,l-phosphinothricin; Duchefa Biochemie, http://www.duchefa-biochemie.com) or by spraying with basta three times at intervals of 8–10 days. PCR, using primers pHanF (5′-TCCCAACTGTAATCAATCC-3′) and pHanR (5′-GACAAGTGATGTGTAAGACG-3′), was performed on selected plants to confirm the presence of the construct.

### Cytological procedures

Meiotic chromosome spreads, FISH and immunolocalization using fresh PMCs from Arabidopsis were carried out as previously described (Higgins *et al*., 2004). Antibodies were used at the following dilutions: anti-ZYP1 (rabbit/rat, 1/500) and anti-ASY1 (rabbit/rat, 1/1000). Immunolocalization on fixed bud material was carried out as described in Chelysheva *et al*. (2010). Anti-AtSMC4 antibody was used at a 1/500 dilution. Chiasma counts on metaphase-I chromosome-spread preparations from PMCs were carried out as described in Sanchez-Moran *et al*. (2002) using *45S* and *5S* rDNA FISH probes. All slides were viewed on a Nikon Eclipse E400 microscope (Nikon, http://www.nikon.com) using cell p soft imaging system software (Olympus, http://www.olympus-global.com). A Hamamatsu ORCA-ER digital camera (Hamamatsu, http://www.hamamatsu.com) was used to capture images.

### Production of antibody

A 241-amino acid region at the C terminus of AtSMC4, corresponding to residues 780–1021, was amplified from Arabidopsis leaf cDNA using an *Nhe*I site incorporated into primer SMC4Ab_F (5′-CCGCTAGCGAACTGGCGAAAAGCCAAAG-3′) and a *Not*I site incorporated into primer SMC4Ab_R (5′-GCGGCCGCTTTCAGATCACAA-3′). The PCR product was ligated into pZero then excised by restriction digestion using *Not*I and *Nhe*I, and ligated into pET21b expression vector (Novagen, now EMD Millipore, http://www.emdmillipore.com). The construct was transformed into *Escherichia coli* BL21 cells (Novagen). The recombinant protein was purified using Ni-NTA resin (Qiagen, http://www.qiagen.com), dialysed against 100 mm NaCl, 2 mm EDTA and 50 mm Tris/HCl, pH 8, to remove urea, and used to raise rabbit polyclonal antiserum (BioGenes GmbH, http://www.biogenes.de). Before use, antiserum was purified using an Immobilized *E. coli* Lysate Kit (Thermo Scientific, http://www.thermoscientific.com).

### Protein extraction and western blotting

Anther proteins were extracted in 150 mm NaCl, 10% glycerol, 2 mm EDTA and Tris-HCl, pH 7.5, containing protease inhibitors (Complete mini EDTA-free tablets; Roche, http://www.roche.com) and insoluble material was removed by centrifugation. Protein samples were separated by SDS-PAGE and western blotted as described by Armstrong *et al*. (2002). Anti-AtSMC4 antibody was used at a dilution of 1/1000.

### Co-immunoprecipitation of condensin complexes

Co-immunoprecipitation of condensin complexes using anti-AtSMC4 antibody was carried out as previously described (Osman *et al*., 2013), using wild-type meiotic buds for protein extraction.

### Statistical procedures

Sail_86_D2 segregation ratios were tested using χ^2^ analysis, variation in chromosome axis lengths and variation in chiasma frequency was tested using the Student's *t*-test. All statistical procedures were carried out using excel (Microsoft, http://www.microsoft.com).

## SUPPORTING INFORMATION

**Figure S1.** Immunolocalization of anti-AtSMC4 pre-immune serum on wild-type Col-0 meiocytes.

**Figure S2.** Gene structures of *AtSMC4* and *Atcap-d3*.

**Figure S3.** Analysis of chromosome axes and synaptonemal complex in condensin-depleted lines at prophase I.

**Figure S4.** Immunolocalization of AtSMC4 (green) on *AtSMC4*^*RNAi-1*^ and wild-type PMCs at metaphase I.

**Figure S5.** Fluorescence *in situ* hybridization using centromere-specific probe pAL1 (green) on pachytene cells of wild-type and condensin-depleted plants.

**Figure S6.** RT-PCR analysis of the *AtCAP-D3* transcript in *Atcap-d3* and wild-type Col-0 plants.

**Figure S7.** Vegetative defects in *Atcap-d3* plants approximately 6 weeks after germination.

**Table S1.** Condensin subunits co-immunoprecipitated with the anti-AtSMC4 antibody.
