# Supplementary figures and images for: The condensin complexes play distinct roles to ensure normal chromosome morphogenesis during meiotic division in Arabidopsis
Source: Plant J. 2014 Jul 26;80(2):255–68. doi: 10.1111/tpj.12628 (PMC4552968; doi:10.1111/tpj.12628)

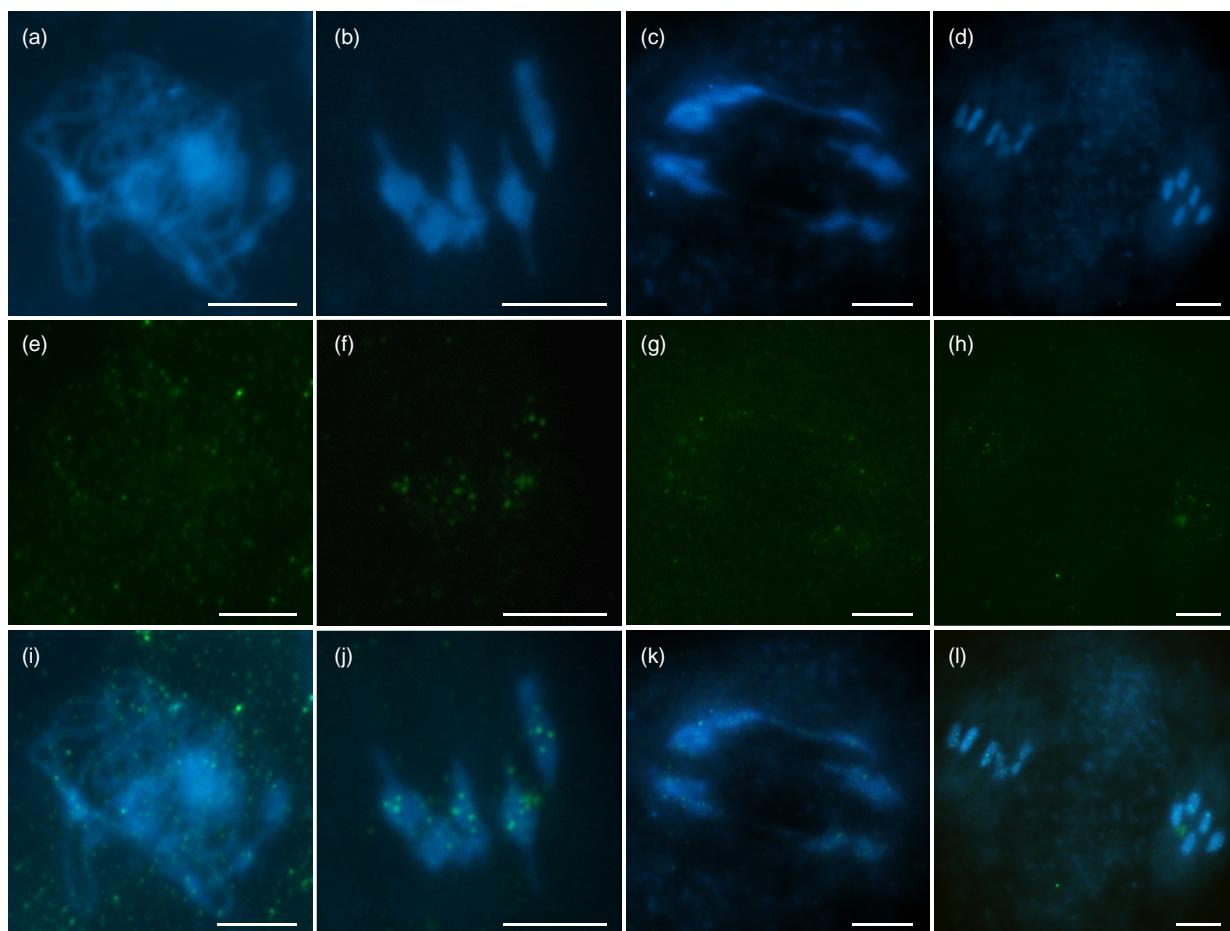

Supplement: Supplementary file 1 — Figure S1. Immunolocalization of anti-AtSMC4 pre-immune serum on wild-type Col-0 meiocytes. [file tpj0080-0255-sd1.pdf]

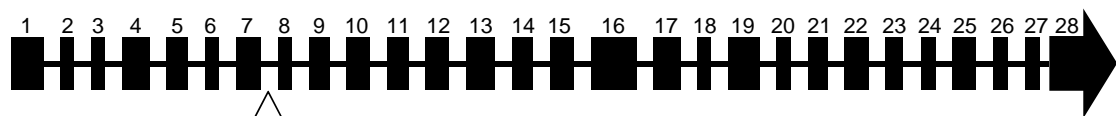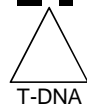

T-DNA

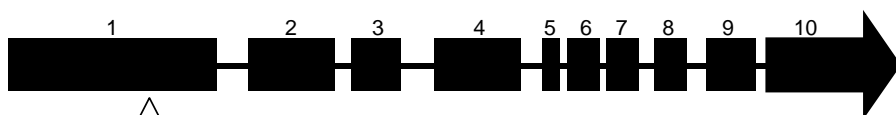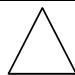

T-DNA

Supplement: Supplementary file 2 — Figure S2. Gene structures of AtSMC4 and Atcap-d3. [file tpj0080-0255-sd2.pdf]

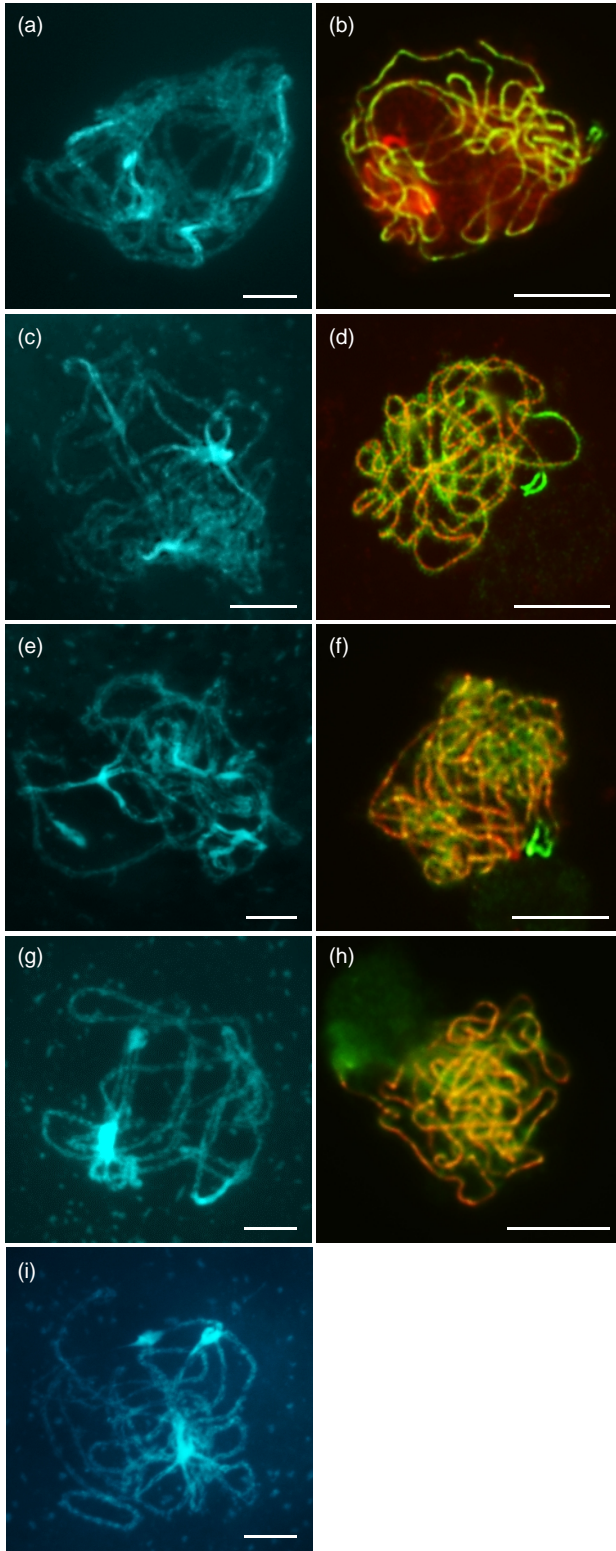

Supplement: Supplementary file 3 — Figure S3. Analysis of chromosome axes and synaptonemal complex in condensin-depleted lines at prophase I. [file tpj0080-0255-sd3.pdf]

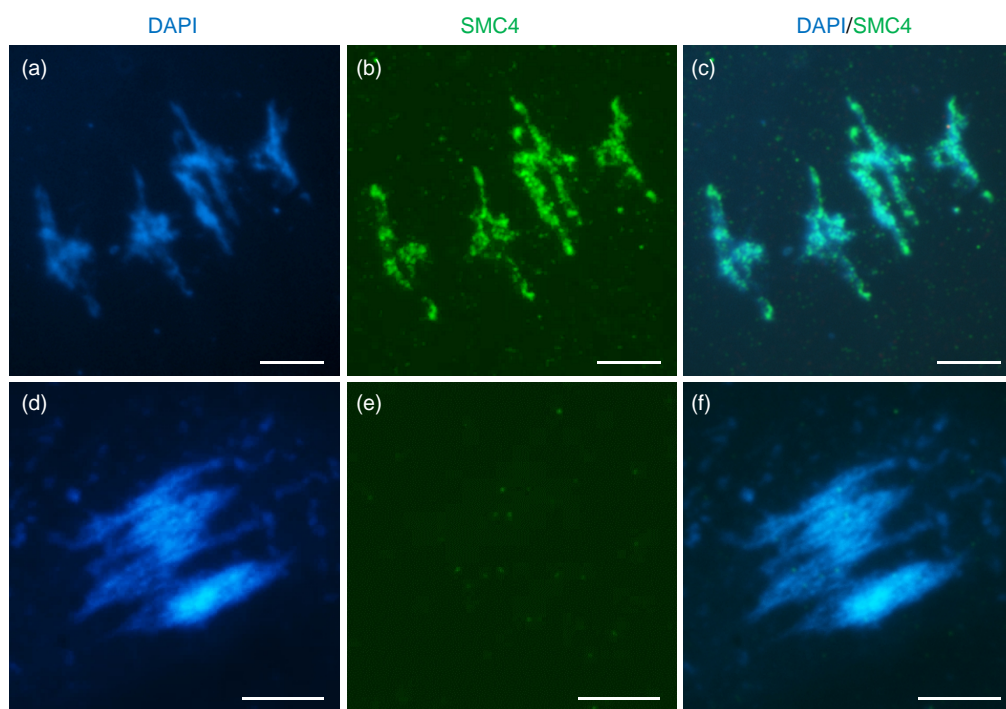

Supplement: Supplementary file 4 — Figure S4. Immunolocalization of AtSMC4 (green) on AtSMC4RNAi-1 and wild-type PMCs at metaphase I. [file tpj0080-0255-sd4.pdf]

DAPI/pAL1

pAL1

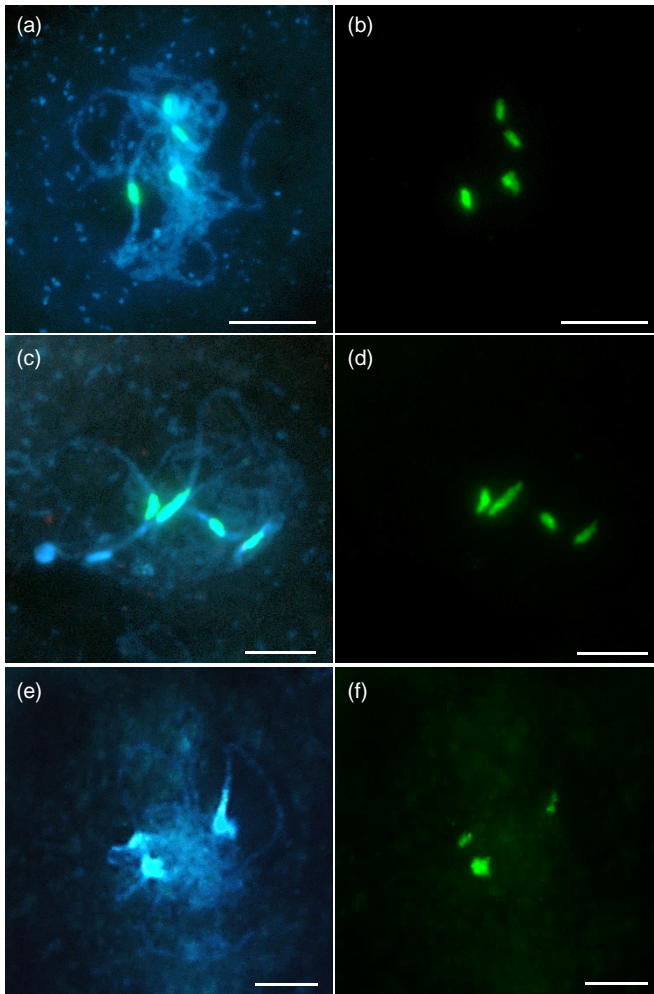

Supplement: Supplementary file 5 — Figure S5. Fluorescence in situ hybridization using centromere-specific probe pAL1 (green) on pachytene cells of wild-type and condensin-depleted plants. [file tpj0080-0255-sd5.pdf]

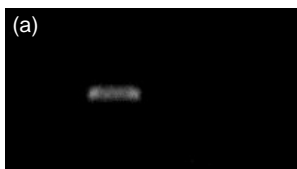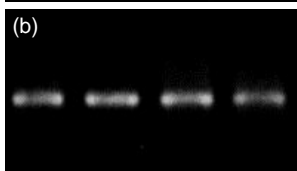

Supplement: Supplementary file 6 — Figure S6. RT-PCR analysis of the AtCAP-D3 transcript in Atcap-d3 and wild-type Col-0 plants. [file tpj0080-0255-sd6.pdf]

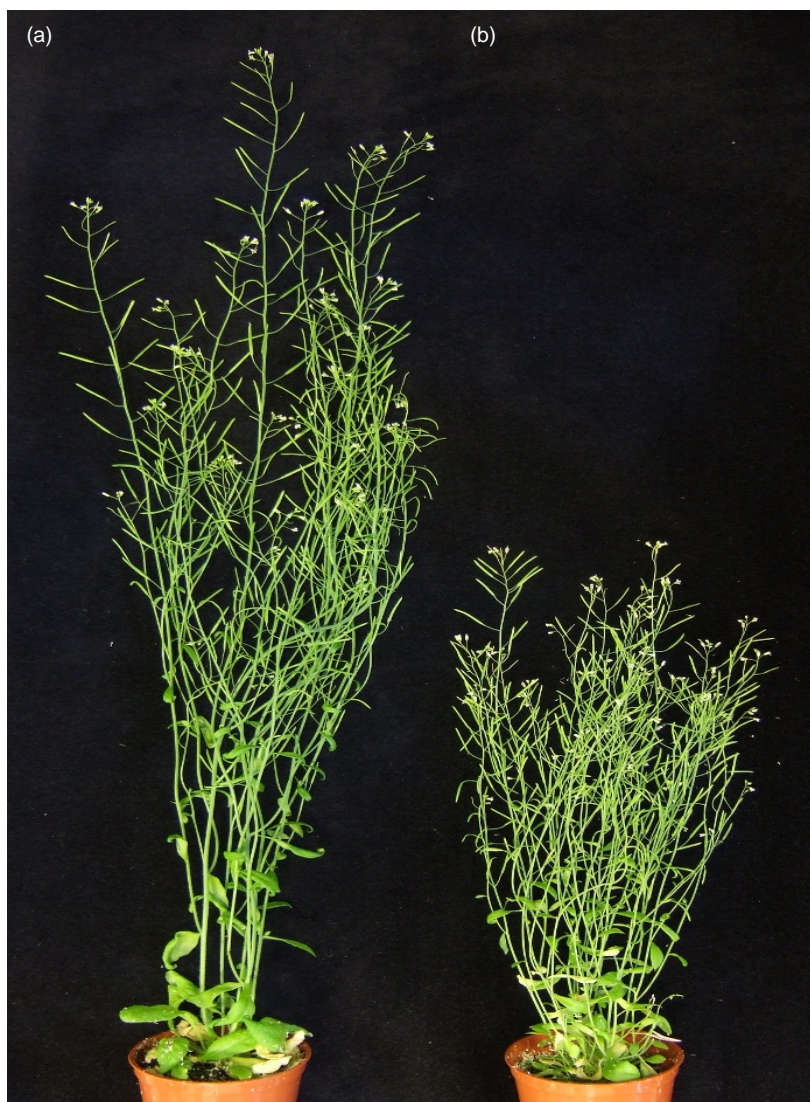

Supplement: Supplementary file 7 — Figure S7. Vegetative defects in Atcap-d3 plants approximately 6 weeks after germination. [file tpj0080-0255-sd7.pdf]
