# Supplementary material for: The condensin complexes play distinct roles to ensure normal chromosome morphogenesis during meiotic division in Arabidopsis
Source: Plant J. 2014 Jul 26;80(2):255–68. doi: 10.1111/tpj.12628 (PMC4552968; doi:10.1111/tpj.12628)
Supplement: Supplementary file 8 — Table S1. Condensin subunits co-immunoprecipitated with the anti-AtSMC4 antibody. [file tpj0080-0255-sd8.docx]

|  | **Condensin subunit** | **Locus** | **Unique spectra** | | **Protein coverage**  **(%)** |
| --- | --- | --- | --- | --- | --- |
|  |  |  | **E1** | **E2** |  |
| SMC proteins: | SMC4 (CAP-C) | At5g48600.2 | 132 | 95 | 59 |
|  | SMC2-1 (CAP-E1) | At5g62410.1 | 93 | 54 | 56 |
|  | SMC2-2 (CAP-E2) | At3g47460.1 | 58 | 22 | 55 |
| Condensin I: | CAP-D2 | At3g57060.1 | 21 | 10 | 17 |
|  | CAP-H | At2g32590 | 17 | 8 | 25 |
|  | CAP-G | At5g37630 | 18 | 8 | 18 |
| Condensin II | CAP-D3 | At4g15890 | 5 | 2 | 5 |
|  | CAP-H2 | At3g16730 | 2 | 1 | 5 |
|  | CAP-G2 | At1g64960 | 1 | 0 | 1 |

**Supplementary Table S1:** Table showing Condensin subunits co-immunoprecipitated with the anti-AtSMC4 antibody. Results from elutions 1 (E1), and 2 (E2) are listed.
