# Supplementary material for: The condensin complexes play distinct roles to ensure normal chromosome morphogenesis during meiotic division in Arabidopsis
Source: Plant J. 2014 Jul 26;80(2):255–68. doi: 10.1111/tpj.12628 (PMC4552968; doi:10.1111/tpj.12628)
Supplement: Supplementary file 9 — Table S1. Condensin subunits co-immunoprecipitated with the anti-AtSMC4 antibody. [file tpj0080-0255-sd9.docx]

**Smith et al. The condensin complexes play distinct roles to ensure normal chromosome morphogenesis during meiotic division in Arabidopsis**

**Legends for Supporting Information**

Table S1

Table showing Condensin subunits co-immunoprecipitated with the anti-AtSMC4 antibody. Results from elutions 1 (E1), and 2 (E2) are listed.

Figure S1

Immunolocalisation of anti-AtSMC4 pre-immune serum on wild-type Col-0 meiocytes. Chromosomes counter-stained with DAPI. a-d = DAPI, e-h = anti-AtSMC4 pre-immune serum, FITC. i=l = DAPI/anti-AtSMC4 pre-immune merge. Scale bar = 5 µm.

Figure S2

Top = *AtSMC4* gene structure the position of the T-DNA insertion in line At5g48600, Sail_86_D2 is indicated by a triangle. Bottom = *Atcapd3* line Sail_826_B06 the position of the T-DNA is indicated by a triangle. Black boxes represent exons, corresponding exon number indicated above. Spaces represent introns. Direction of gene corresponds to direction of arrow head.

Figure S3

Analysis of chromosome axes and SC in condensin depleted lines at prophase I. a, c, e, g and i = DAPI stained (blue) chromosome spreads of late zygotene/pachytene nuclei. b, d, f and h = immunolocalisation of the axis protein AtASY1 (green) and SC transverse filament protein AtZYP1 (red) on PMCs. a and b = wild-type, c and d = *AtSMC4/Atsmc4*, e and f = *AtSMC4^RNAi-1^*, g and h = *Atcap-d3*, i = *AtCAP-D2^RNAi^*. Scale bar = 5 µm.

Figure S4

Immunolocalisation of AtSMC4 (green) on *AtSMC4^RNAi-1^* and wild-type PMCs at metaphase I. Chromosomes counter-stained with DAPI (Blue). **a-c** = Wild-type; **d-f** = *AtSMC4^RNAi-1^*. Scale bar = 5 µm.

Figure S5

Fluorescence *in situ* hybridisation using centromere specific probe pAL1 (green) on pachytene cells of wild-type and condensin depleted plants. a and b = wild-type. c and d = *SMC4^RNAi-1^*, e and f = *SMC4^RNAi-2^*. Scale bar 5 µm.

Figure S6

RT-PCR analysis of the *AtCAP-D3* transcript in *Atcap-d3* and wild-type Col-0 plants: (a) *AtCAP-D3* expression. (b) *GAPD* loading control. Lanes left to right: wild-type leaf, wild-type bud, *Atcap-d3* leaf, *Atcap-d3* bud.

Figure S7

Vegetative defects in *Atcap-d3* plants approximately 6 weeks after germination. a = wild-type. b = *Atcap-d3.*
